# Hospitalizations for Food-Induced Anaphylaxis Between 2016 and 2021: Population-Based Epidemiologic Study

**DOI:** 10.2196/57340

**Published:** 2024-08-27

**Authors:** Rodrigo Jimenez-Garcia, Ana Lopez-de-Andres, Valentin Hernandez-Barrera, Jose J Zamorano-Leon, Natividad Cuadrado-Corrales, Javier de Miguel-Diez, Jose L del-Barrio, Ana Jimenez-Sierra, David Carabantes-Alarcon

**Affiliations:** 1 Department of Public Health & Maternal and Child Health Faculty of Medicine Universidad Complutense de Madrid Madrid Spain; 2 Preventive Medicine and Public Health Teaching and Research Unit Health Sciences Faculty Universidad Rey Juan Carlos Alcorcon Spain; 3 Respiratory Care Department Hospital General Universitario Gregorio Marañón Madrid Spain; 4 Faculty of Medicine Universidad San Pablo CEU Madrid Spain

**Keywords:** food-induced anaphylaxis, epidemiology, hospitalizations, in-hospital mortality

## Abstract

**Background:**

Food-induced anaphylaxis (FIA) is a major public health problem resulting in serious clinical complications, emergency department visits, hospitalization, and death.

**Objective:**

This study aims to assess the epidemiology and the trends in hospitalizations because of FIA in Spain between 2016 and 2021.

**Methods:**

An observational descriptive study was conducted using data from the Spanish National Hospital discharge database. Information was coded based on the *International Classification of Diseases, Tenth Revision*. The study population was analyzed by gender and age group and according to food triggers, clinical characteristics, admission to the intensive care unit, severity, and in-hospital mortality. The annual incidence of hospitalizations because of FIA per 100,000 person-years was estimated and analyzed using Poisson regression models. Multivariable logistic regression models were constructed to identify which variables were associated with severe FIA.

**Results:**

A total of 2161 hospital admissions for FIA were recorded in Spain from 2016 to 2021. The overall incidence rate was 0.77 cases per 100,000 person-years. The highest incidence was found in those aged <15 years (3.68), with lower figures among those aged 15 to 59 years (0.25) and ≥60 years (0.29). Poisson regression showed a significant increase in incidence from 2016 to 2021 only among children (3.78 per 100,000 person-years vs 5.02 per 100,000 person-years; *P*=.04). The most frequent food triggers were “milk and dairy products” (419/2161, 19.39% of cases) and “peanuts or tree nuts and seeds” (409/2161, 18.93%). Of the 2161 patients, 256 (11.85%) were hospitalized because FIA required admission to the intensive care unit, and 11 (0.51%) patients died in the hospital. Among children, the most severe cases of FIA appeared in patients aged 0 to 4 years (40/99, 40%). Among adults, 69.4% (111/160) of cases occurred in those aged 15 to 59 years. Multivariable logistic regression showed the variables associated with severe FIA to be age 15 to 59 years (odds ratio 5.1, 95% CI 3.11-8.36), age ≥60 years (odds ratio 3.87, 95% CI 1.99-7.53), and asthma (odds ratio 1.71,95% CI 1.12-2.58).

**Conclusions:**

In Spain, the incidence of hospitalization because of FIA increased slightly, although the only significant increase (*P*=.04) was among children. Even if in-hospital mortality remains low and stable, the proportion of severe cases is high and has not improved from 2016 to 2021, with older age and asthma being risk factors for severity. Surveillance must be improved, and preventive strategies must be implemented to reduce the burden of FIA.

## Introduction

### Background

Food allergy (FA) is a major public health issue globally, affecting approximately 10% of adults and 8% of children and being more common in urban areas and high-income countries [1]. A significant proportion of patients with FA experience food-induced anaphylaxis (FIA) [2-9].

FIA is a severe allergic reaction that can occur immediately after ingestion of food, and it can lead to serious complications, emergency department visits, hospitalization, and death [2-8].

Furthermore, FIA has significant effects on health-related quality of life for both patients and their families and generates a considerable emotional, social, and financial burden [9-11].

Among 5587 patients with FAs included in the Food Allergy Research and Education Patient Registry, 42% experienced >1 reaction per year and almost half of all patients reported a previous FIA (46%) [9].

The lifetime prevalence of FIA is <0.5%, with children and young adults being the most frequently affected groups [3,8,11]. In the United States, the incidence of FIA in the population rose from 86.3 per 100,000 person-years in 2004 to 239.2 per 100,000 person-years in 2016 [12].

Mortality is estimated to range from 0.03 to 0.3 per million inhabitants per year, figures significantly lower than those found for other causes of anaphylaxis [3,8,11,13,14].

However, the incidence of near-fatal FIA, defined as that requiring admission to the intensive care unit (ICU), is calculated to be 10 times higher [15].

Several studies have assessed the importance of triggers and other variables associated with severity and death in patients who experience FIA and report potential risk factors to include milk products, peanuts, older age, male gender, uncontrolled asthma, physical activity, drugs, and alcohol [5,6,8,13,16-20]. Gaps in the management during prehospital emergency care (not to use the adrenaline autoinjectors [AAIs] and nonmedical emergency vehicles) and risky behavior, such as lack of vigilance related to allergen avoidance for known allergies and noncompliance with regard to carrying the first aid emergency kit at all times, have been pointed out in patients with FA who experienced a severe FIA reaction [19,20].

Recent studies conducted in several countries have shown an increase in the incidence of hospitalization because of FIA, apparently temporarily interrupted by the effect of the COVID-19 pandemic and driven mainly by increasingly frequent reports of the condition in children [3,5,8,17,18,21-24].

However, this trend seems not to be associated with an increase in the incidence of severe or fatal reactions [5,10,21] but more with improved early diagnosis and treatment, including greater availability and use of AAIs, which could account for the stable trend in the severity and mortality in patients with FIA in many studies [5,10,18,21].

Most cases of FIA are caused by cow’s milk, egg, peanut, tree nuts, fish, shellfish, wheat, and soy, although geographic differences in feeding patterns and diets markedly affect the frequency of food triggers in each region [3,4,6-8,17,18,21,25].

In Spain, the first cause was drugs (42.48%), the second was unknown causes (30.59%), and FIA was the third most frequent cause (19.64%). While the incidence of admissions for FIA during this period rose in all age groups, the most significant increases were observed among children [26]. The 3 most common triggers were milk, eggs, and fish [26].

Research on the epidemiology of FIA enables us to monitor the effect of FA on public health and to improve the implementation of key preventive strategies [18,27]. However, nationally representative data to estimate the frequency of hospitalizations occurring in the Spanish population are scarce [26,28].

In Spain, the National Health System is a publicly funded health insurance system offering universal coverage to individuals at no cost. It is fully financed by the general tax fund, thereby ensuring nationwide data availability for acute diseases such as FIA. Real-world health care data sources, including hospital discharge databases, facilitate our understanding of trends in incidence and morbidity and the identification of potential risk factors for severe or fatal FIA [5,7,10,11,17,18,21,22,26-28].

### Objectives

In this study, we used a national representative hospital discharge database to investigate changes in hospitalization because of FIA in Spain between 2016 and 2021. We also analyzed food triggers, clinical characteristics, and outcomes of hospitalization among children and adults who were admitted with FIA. In addition, we analyzed factors associated with the severity of FIA.

## Methods

### Study Design

To achieve the proposed objectives, we designed an observational descriptive study based on an analysis of national hospital data to evaluate time trends in the incidence of FIA following a methodology described elsewhere [5,17,22,26]. Likewise, to identify the factors associated with the severity of FIA, we conducted a retrospective observational cohort study reproducing work carried out in Spain and in other countries [17,28].

### Setting and Participants

The study was conducted using hospital discharge data collected in the *Conjunto Minimo Basico de Datos* (CMBD); this is the name for the Spanish National Registry of hospital discharges. This information system is mandatory for public and private hospitals in Spain, and details can be found elsewhere [29,30]. Briefly, the database includes basic sociodemographic information, a primary diagnosis, and up to 19 secondary diagnoses. As per the CMBD methodology, the primary or main diagnosis is the condition that, following investigation, was identified as the primary reason for the patient’s hospital admission. Secondary diagnoses encompass all risk factors or preexisting conditions present at admission or emerging during hospitalization that the treating physician deems to potentially impact the patient’s treatment, need for procedures, or progress [29,30]. It also includes a maximum of 20 therapeutic or diagnostic procedures performed on the patient hospitalized for FIA. Finally, the outcome of the hospital stay and its duration are recorded. Information is coded based on the *International Classification of Diseases, Tenth Revision* (*ICD-10*).

We selected patients admitted to any medical or surgical departments, including ICUs, for a minimum of 24 hours. Patients treated solely in emergency departments without being transferred to a hospital room are not included in the database.

A total of 2 strategies were used to identify cases of FIA. First, patients with FIA as their primary diagnostic *ICD-10* code were selected (refer to Multimedia Appendix 1 for codes). Second, we included patients with a secondary diagnostic *ICD-10* code for FIA in any field and a primary diagnostic code that corresponded to a symptom, sign, organ, system, or procedure that was closely related to an anaphylactic reaction, as reported elsewhere [26,31].

These codes and their frequencies are shown in Multimedia Appendix 2.

### Variables

The outcome variables were the estimated annual incidence of hospitalizations due to FIA per 100,000 inhabitants stratified by gender and age group. Population data were obtained from the Spanish National Institute of Statistics [32]. We also created the outcome variable *severe FIA*, defined as severe episodes requiring admission to the ICU or fatal episodes.

The study population was described and analyzed by gender and age group (0-14 years, 15-59 years, and ≥60 years) and according to age status (children vs adults).

The food groups responsible for anaphylaxis were categorized as “unspecified food,” “peanuts or tree nuts and seeds,” “shellfish (crustaceans) or other fish,” “fruits and vegetables,” “milk and dairy products,” “eggs,” and “other food products or food additives.”

The clinical variables and diagnostic procedures analyzed with their corresponding *ICD-10* codes are shown in Multimedia Appendix 1. Age, gender, culprit foods, and the clinical variables studied were analyzed as possible risk factors for severe FIA.

### Statistical Analysis

The trend in the incidence of FIA hospital admissions between 2016 and 2021 was analyzed using Poisson regression models adjusted for age and gender, as needed. Poisson regression is applied to model counts or events that occur randomly over a period in a fixed space. It is often useful when the probability of an event is very small and the population is very large, as occurs in the case of hospitalization for FIA. Poisson regression has previously been applied to assess the time trend in hospital admission with FIA and any type of anaphylaxis using national hospital data [5,17,22,26].

The normal distribution of continuous variables, such as age, was assessed using the Kolmogorov-Smirnov test. With a *P* value of .04, we concluded that this variable followed a normal distribution within our population.

The quantitative variable was expressed as mean with SD, and bivariate analysis was conducted with 2-tailed *t* test. Quantitative variables were analyzed using the Fisher exact test and expressed as percentages.

Bivariate linear regression was used to test the linear temporal trends for mean age from 2016 to 2021, as this was a normally distributed continuous variable. The Cochran-Armitage test was applied to assess trends for qualitative variables.

Multivariable logistic regression models were constructed following the recommendations by Hosmer et al [33] to identify which variables were independently associated with severe FIA. Multivariable logistic regression models are commonly applied to identify variables associated with binary end points, such as the presence or absence of disease in diagnostic models, or short-term prognostic events, such as in-hospital mortality (IHM) or severity. For logistic regression, all participants had to have been followed up for the duration of the study period [33]. This statistical method has been applied elsewhere to assess predictors of severity in patients hospitalized for anaphylaxis [17,28].

All statistical analyses were run in STATA (version 14.0; StataCorp LLC). A *P* value of <.05 (2-tailed) was considered statistically significant.

### Ethical Considerations

The CMBD is the property of the Spanish Ministry of Health and is made available free of charge upon request [34]. We requested the data needed for this investigation following the Spanish Ministry of Health protocol and received permission to use the CMBD database [34]. No compensation was provided to the participants.

Given the registry’s anonymous nature, individual written consent from patients or approval from an institutional review board was not needed in accordance with the Spanish legislation [35,36].

## Results

### Time Trends in the Incidence of Hospitalization Due to FIA

As presented in Table 1, there were a total of 2161 admissions for FIA in Spain between 2016 and 2021. By year, the minimum was observed in 2020 (305/2161, 14.11%) and the maximum was observed in the last year studied, that is, 2021 (427/2161, 19.76%).

**Table 1:** Incidence and characteristics of hospital admissions with a diagnosis of food-induced anaphylaxis in Spain between 2016 and 2021.

Characteristics		2016 (n=371)	2017 (n=336)	2018 (n=364)	2019 (n=358)	2020 (n=305)	2021 (n=427)	Total (N=2161)	*P* value for time trend
Women, n (%)		133 (35.85)	151 (44.94)	153 (42.03)	161 (44.97)	112 (36.72)	176 (41.22)	886 (41)	.051
**Incidence per 100,000 inhabitants, n**									
	Total	0.80	0.72	0.78	0.76	0.64	0.90	0.77	.42
	Men	1.04	0.81	0.92	0.85	0.83	1.08	0.92	.71
	Women	0.56	0.64	0.64	0.67	0.46	0.73	0.62	.43
**Age group (y), n (%)**									
	0-14	264 (71.16)	228 (67.86)	259 (71.15)	236 (65.92)	212 (69.51)	336 (78.69)	1535 (71.03)	.002
	15-59	71 (19.14)	83 (24.7)	63 (17.31)	78 (21.79)	64 (20.98)	67 (15.69)	426 (19.71)	.002
	≥60	36 (9.7)	25 (7.44)	42 (11.54)	44 (12.29)	29 (9.51)	24 (5.62)	200 (9.25)	.002
**Age group (y), incidence per 100,000 inhabitants**									
	0-14	3.78	3.27	3.73	3.43	3.10	5.02	3.68	.04
	15-59	0.25	0.29	0.22	0.28	0.22	0.24	0.25	.65
	≥60	0.32	0.22	0.36	0.37	0.24	0.19	0.29	.53
Age (y), mean (SD)		17.9 (22.6)	18.0 (21.3)	17.9 (23.2)	20.4 (23.8)	18.3 (23.1)	13.5 (18.6)	17.5 (22.1)	.001
**Foods responsible for anaphylaxis, n (%)**									
	Unspecified food	36 (9.7)	37 (11.01)	42 (11.54)	38 (10.61)	38 (12.46)	32 (7.49)	223 (10.32)	.29
	Peanuts and tree nuts and seeds	79 (21.29)	66 (19.64)	71 (19.51)	70 (19.55)	42 (13.77)	81 (18.97)	409 (18.93)	.22
	Shellfish (crustaceans) and other fish	42 (11.32)	34 (10.12)	42 (11.54)	49 (13.69)	29 (9.51)	22 (5.15)	218 (10.09)	.003
	Fruits and vegetables	21 (5.66)	32 (9.52)	34 (9.34)	29 (8.1)	33 (10.82)	25 (5.85)	174 (8.05)	.06
	Milk and dairy products	72 (19.41)	72 (21.43)	75 (20.6)	58 (16.21)	61 (20)	81 (18.97)	419 (19.39)	.59
	Eggs	19 (5.12)	20 (5.95)	21 (5.77)	13 (3.63)	15 (4.92)	26 (6.09)	114 (5.28)	.68
	Other food products or food additives	43 (11.59)	36 (10.71)	33 (9.07)	35 (9.78)	32 (10.49)	23 (5.39)	202 (9.35)	.04
**Hospitalization, n (%)**									
	Invasive mechanical ventilation	6 (1.62)	14 (4.17)	12 (3.3)	22 (6.15)	10 (3.28)	7 (1.64)	71 (3.25)	.005
	Noninvasive mechanical ventilation	0 (0)	4 (1.19)	2 (0.55)	5 (1.4)	2 (0.66)	4 (0.94)	17 (0.75)	.32
	Admission to ICU^a^	33 (8.89)	50 (14.88)	48 (13.19)	61 (17.04)	32 (10.49)	32 (7.49)	256 (11.85)	<.001
	IHM^b^	0 (0)	1 (0.3)	4 (1.1)	3 (0.84)	2 (0.66)	1 (0.23)	11 (0.51)	.28
	Severe anaphylaxis^c^	33 (8.89)	50 (14.88)	48 (13.19)	62 (17.32)	33 (10.82)	33 (7.73)	259 (11.99)	<.001

^a^ICU: intensive care unit.

^b^IHM: in-hospital mortality.

^c^Severe anaphylaxis included IHM and admission to ICU.

The overall incidence rate from 2016 to 2021 was 0.77 cases per 100,000 person-years. The highest incidence was found in the younger age group (3.68), with lower values among those aged 15 to 59 years (0.25) and those aged ≥60 years (0.29).

Poisson regression showed a significant increase in incidence from 2016 to 2021 only among children (3.78 per 100,000 vs 5.02 per 100,000; *P*=.04).

By gender, women accounted for 40.99% (886/2161) of all FIA admissions, with no significant changes in this percentage during the study period (*P*=.051). Similarly, Poisson regression analysis revealed no variations in incidence in women or men.

Between 2016 and 2021, a total of 71.03% (1535/2161) of hospitalized patients were children (aged 0-14 years), followed in frequency by patients aged 15 to 59 years (426/2161, 19.71%); the lowest percentage was recorded for patients aged ≥60 years (200/2161, 9.25%).

The mean age of patients with FIA decreased from 17.9 (SD 2.45) years in 2016 to 13.5 (SD 1.96) years in 2021 (*P*=.001).

### Food as a Trigger, Clinical Characteristics, and Outcomes of Hospitalizations Due to FIA Among Children and Adults

Analysis of the causative foods revealed the 2 most frequent foods to be “milk and dairy products” (419/2161, 19.39% of cases) and “peanuts and tree nuts and seeds” (409/2161, 18.93%). The prevalence of both triggers remained stable over time. A significant decrease was observed in “shellfish (crustaceans) and other fish,” falling from 11.3% (42/371) in 2016 to 5.6% (22/427) in 2021 (*P*=.003).

Invasive mechanical ventilation was recorded in 3.29% (71/2161) of patients with FIA, while noninvasive ventilation was recorded in 0.79% (17/2161). In total, 11.85% (256/2161) of patients were admitted to the ICU and 0.51% (11/2161) of patients died in the hospital; therefore, 11.99% (259/2161) of patients were classified as having severe FIA. The proportion of severe cases decreased from 2016 to 2021 (33/371, 8.9% vs 33/427, 7.7%; *P*<.001).

The frequency of food as a trigger differed significantly between children and adults. The most frequent triggers in children were “milk and dairy products” (405/1200, 33.75%), followed by “peanuts and tree nuts and seeds” (307/1200, 25.58%) and “eggs” (113/1200, 9.42%). In patients aged ≥15 years, “shellfish (crustaceans) and other fish” was recorded in 30.2% (169/559) of patients and “unspecified food” was recorded in 18.3% (102/559) of patients.

Table 2 compares the characteristics of patients hospitalized for FIA between 2016 and 2021 according to gender and age group. When comparing women with men, we find no significant differences for any of the variables studied (all *P*>.05). Similarly, repeating the analysis by gender and then by age group reveals no significant associations (all *P*>.05).

**Table 2:** Characteristics of hospital admissions with a diagnosis of food-induced anaphylaxis in Spain between 2016 and 2021, according to gender and age group (N=2161).

Characteristics of hospital admissions	0-14 years, n (%)			15-59 years, n (%)			≥60 years, n (%)			All age groups, n (%)		
	Men (n=951)	Women (n=584)	*P* value	Men (n=222)	Women (n=204)	*P* value	Men (n=102)	Women (n=98)	*P* value	Men (n=1275)	Women (n=886)	*P* value
Unspecified food	74 (7.78)	47 (8.05)	.85	28 (12.61)	34 (16.67)	.24	21 (20.59)	19 (19.39)	.83	123 (9.65)	100 (11.29)	.22
Peanuts and tree nuts and seeds	208 (21.67)	109 (18.66)	.13	35 (15.77)	42 (20.59)	.20	7 (6.86)	8 (8.16)	.73	250 (19.61)	159 (17.05)	.33
Shellfish (crustaceans) and other fish	33 (3.47)	16 (2.74)	.43	47 (21.17)	42 (20.59)	.88	38 (37.25)	42 (42.86)	.42	118 (9.25)	100 (11.29)	.12
Fruits and vegetables	57 (5.99)	29 (4.97)	.40	32 (14.41)	27 (13.24)	.72	16 (15.69)	13 (13.27)	.63	105 (8.24)	69 (7.79)	.71
Milk and dairy products	223 (23.45)	182 (31.16)	.09	7 (3.15)	7 (3.43)	.87	0 (0)	0 (0)	—^d^	230 (18.04)	189 (21.33)	.06
Eggs	75 (7.89)	38 (6.51)	.31	1 (0.45)	0 (0)	.34	0 (0)	0 (0)	—	76 (5.96)	38 (4.29)	.09
Other food products or food additives	82 (8.62)	27 (4.62)	.06	43 (19.37)	27 (13.24)	.09	11 (10.78)	12 (12.24)	.75	136 (10.67)	66 (7.45)	.06
Invasive mechanical ventilation	6 (0.63)	6 (1.03)	.39	25 (11.26)	17 (8.33)	.31	12 (11.76)	5 (5.1)	.09	43 (3.37)	28 (3.16)	.79
Noninvasive mechanical ventilation	5 (0.53)	6 (1.03)	.26	3 (1.35)	3 (1.47)	.92	0 (0)	0 (0)	—	8 (0.63)	9 (1.02)	.31
Admission to ICU^a^	54 (5.68)	44 (7.53)	.15	57 (25.68)	54 (26.47)	.85	26 (25.49)	21 (21.43)	.50	137 (10.75)	119 (13.43)	.06
IHM^b^	0 (0)	3 (0.51)	.27	1 (0.45)	1 (0.49)	.95	4 (3.92)	2 (2.04)	.44	5 (0.39)	6 (0.68)	.36
Severe anaphylaxis^c^	54 (5.68)	45 (7.71)	.12	57 (25.68)	54 (26.47)	.85	28 (27.45)	21 (21.43)	.32	139 (10.9)	120 (13.54)	.06

^a^ICU: intensive care unit.

^b^IHM: in-hospital mortality.

^c^Severe anaphylaxis included IHM and admission to ICU.

^d^Not applicable.

As presented in Table 2, the use of invasive mechanical ventilation, admission to the ICU, and IHM increased with the age of the patients hospitalized for FIA. Severe anaphylaxis was recorded in 99 (6.5%) of 1523 children; this proportion increased to 25.9% (111/429) among those aged 15 to 59 years and to 24.5% (49/200) among those aged ≥60 years.

Of the 3 children who died of FIA, the food involved was identified in all of them, with 1 case each for “peanuts and tree nuts and seeds,” “fruits and vegetables,” and “milk and dairy products” (Table 3). Among adults, only 3 (38%) of the 8 patients had a coded food trigger, 2 (25%) patients with “shellfish (crustaceans) and other fish” and 1 (13%) with “fruits and vegetables.”

**Table 3:** Cause of fatal food-induced anaphylaxis by a trigger in children (aged 0-14 years) and adults (aged ≥15 years) in Spain between 2016 and 2021.

Food trigger	Children (aged 0-14 years; n=3), n (%)	Adults (aged ≥15 years; n=8), n (%)	Total (n=11), n (%)
Unspecified food	0 (0)	3 (38)	3 (27)
Peanuts and tree nuts and seeds	1 (33)	0 (0)	1 (9)
Shellfish (crustaceans) and other fish	0 (0)	2 (25)	2 (18)
Fruits and vegetables	1 (33)	1 (13)	2 (18)
Milk and dairy products	1 (33)	0 (0)	1 (9)
Eggs	0 (0)	0 (0)	0 (0)
Other food products or food additives	0 (0)	2 (25)	2 (18)

Table 4 shows the clinical characteristics of patients hospitalized for FIA according to age. In children, the most frequently coded pathology was atopic dermatitis, followed by asthma, with the prevalence of all other diseases being <1%. Among adults, the most frequent conditions were hypertension, asthma, diabetes mellitus, and obesity. As for symptoms associated with FIA according to age status (children vs adults), children more frequently presented nausea or vomiting, abdominal pain, and urticaria and less frequently presented acute respiratory failure, hypotension, and syncope.

**Table 4:** Characteristics, chronic conditions, specific signs, symptoms, and hospital outcomes of hospital admissions with a diagnosis of food-induced anaphylaxis in Spain between 2016 and 2021, according to age groups.

Variable	Children (aged 0-14 years; n=1535), n (%)	Adults (aged ≥15 years; n=626), n (%)	*P* value
Smoking	0 (0)	82 (13.1)	<.001
Obesity	3 (0.20)	58 (9.27)	<.001
GERD^a^	13 (0.85)	8 (1.28)	.35
Chronic rhinitis	1 (0.07)	4 (0.64)	.01
Atopic dermatitis	224 (14.59)	15 (2.4)	<.001
Anxiety	0 (0)	25 (3.99)	<.001
Depression	0 (0)	13 (2.08)	<.001
COPD^b^	0 (0)	32 (5.11)	<.001
Asthma	197 (12.83)	115 (18.37)	.001
Hypertension	0 (0)	156 (24.92)	<.001
Ischemic heart disease	0 (0)	8 (1.28)	<.001
Atrial fibrillation	0 (0)	29 (4.63)	<.001
Hypothyroidism	4 (0.26)	27 (4.31)	<.001
Hyperthyroidism	0 (0)	6 (0.96)	<.001
Diabetes mellitus	0 (0)	92 (14.7)	<.001
Hypotension	18 (1.17)	25 (3.99)	<.001
Syncope or collapse	4 (0.26)	13 (2.08)	<.001
Nausea or vomiting	157 (10.23)	9 (1.44)	<.001
Abdominal pain	83 (5.41)	11 (1.76)	<.001
Acute respiratory failure	47 (3.06)	61 (9.74)	<.001
Urticaria	87 (5.67)	19 (3.04)	.01

^a^GERD: gastroesophageal reflux disease.

^b^COPD: chronic obstructive pulmonary disease.

### Variables Associated With Severe FIA Among Hospitalized Children and Adults

The characteristics of children and adults with severe FIA in Spain from 2016 to 2021 are presented in Table 5. Among children, most cases of severe FIA were in the age groups of 0 to 4 years (40/99, 40%) and 5 to 9 years (39/99, 39%). Among adults, 69.4% (111/160) of cases occurred in those aged 15 to 59 years. When comparing children and adults, we found significant differences between the most frequently identified foods, namely, “milk and dairy products” (41/99, 41% in children vs 5/160, 3.1% in adults; *P*<.001), followed by “peanuts and tree nuts and seeds” (27/99, 27% in children vs 26/160, 16.2% in adults; *P*=.03). However, among adults, the most frequently identified foods were “shellfish (crustaceans) and other fish” (48/160, 30% in adults vs 1/99, 1% in children; *P*<.001) and “unspecified food” (27/160, 16.9% in adults vs 8/99, 8% in children; *P*<.001).

**Table 5:** Characteristics of children and adults with severe anaphylaxis during a hospital admission with a diagnosis of food-induced anaphylaxis in Spain between 2016 and 2021, according to age.

Characteristics		Children (aged 0-14 years; n=99), n (%)	Adults (aged ≥15 years; n=160), n (%)	*P* value
**Age group (y)**				
	0-4	40 (40.4)	—^a^	<.001
	5-9	39 (39.4)	—	<.001
	10-14	20 (20.2)	—	<.001
	15-59	—	111 (69.4)	<.001
	≥60	—	49 (30.6)	<.001
**Gender**				
	Men	54 (54.5)	85 (53.1)	.82
	Women	45 (45.4)	75 (46.9)	.82
**Year**				
	2016	12 (12.1)	21 (13.1)	.16
	2017	23 (23.2)	27 (16.9)	.16
	2018	24 (24.2)	24 (15)	.16
	2019	20 (20.2)	42 (26.3)	.16
	2020	12 (12.1)	21 (13.1)	.16
	2021	8 (8.1)	25 (15.6)	.16
**Food triggers**				
	Unspecified food	8 (8.1)	27 (16.9)	.04
	Peanuts and tree nuts and seeds	27 (27.3)	26 (16.3)	.03
	Shellfish (crustaceans) and other fish	1 (1)	48 (30)	<.001
	Fruits and vegetables	2 (2)	23 (14.4)	.001
	Milk and dairy products	41 (41.4)	5 (3.1)	<.001
	Eggs	6 (6.1)	1 (0.6)	.009
	Other food products or food additives	9 (9.1)	24 (15)	.17
**Hospitalization**				
	Smoking	0 (0)	21 (13.1)	<.001
	Obesity	0 (0)	18 (11.3)	.001
	GERD^b^	1 (1)	1 (0.6)	.73
	Chronic rhinitis	0 (0)	1 (0.6)	.41
	Atopic dermatitis	8 (8.1)	2 (1.3)	.006
	Anxiety	0 (0)	10 (6.3)	.01
	Depression	0 (0)	3 (1.9)	.17
	COPD^c^	0 (0)	10 (6.3)	.01
	Asthma	17 (17.2)	41 (25.6)	.11
	Hypertension	0 (0)	44 (27.5)	<.001
	Ischemic heart disease	0 (0)	2 (1.3)	.26
	Atrial fibrillation	0 (0)	12 (7.5)	.005
	Hypothyroidism	0 (0)	3 (1.9)	.17
	Hyperthyroidism	0 (0)	1 (0.6)	.43
	Diabetes mellitus	0 (0)	25 (15.6)	<.001
	Hypotension	5 (5)	8 (5)	.98
	Syncope or collapse	1 (1)	1 (0.6)	.73
	Nausea or vomiting	6 (6.1)	2 (1.3)	.03
	Abdominal pain	0 (0)	1 (0.6)	.43
	Acute respiratory failure	10 (10.1)	36 (22.5)	.01
	Urticaria	6 (6.1)	4 (2.5)	.14

^a^Not applicable.

^b^GERD: gastroesophageal reflux disease.

^c^COPD: chronic obstructive pulmonary disease.

The results of the multivariable logistic regression models constructed to identify the variables associated with severe FIA in children, adults, and the full study population are presented in Multimedia Appendix 3. The only variable associated with severe FIA in all 3 models was acute respiratory failure. The variables associated with severe FIA were hypotension in children (odds ratio [OR] 4.17, 95% CI 1.38-12.6) and ischemic heart disease in adults (OR 2.25, 95% CI 1.48-3.68). In the full study population model with children as the reference, the age groups associated with severe FIA were 15 to 59 years (OR 5.1, 95% CI 3.11-8.36) and ≥60 years (OR 3.87, 95% CI 1.99-7.53). Finally, asthma was associated with severe FIA when children and adults were analyzed together (OR 1.71, 95% CI 1.12-2.58). In none of the models did the prevalence of severe FIA change significantly over time after adjusting for study covariates.

## Discussion

### Principal Findings

In this retrospective study performed over a 6-year period, we investigated the characteristics and outcomes of hospital admissions attributable to FIA. Our findings indicate a stable trend (*P*=.42) with a small but significant rise in hospital admissions for FIA among children. However, neither the number of deaths nor the proportion of severe cases has varied over time. Older age and previous asthma were associated with severe FIA.

### Comparison With Prior Work

In the United Kingdom, 30,700 hospital admissions for FIA were recorded from 1998 to 2018. The overall incidence increased over the study period from 1.23 to 4.04 admissions per 100,000 person-years [5]. This overall rate was much higher than the rate recorded in our study (0.77 cases per 100,000 person-years from 2016 to 2021). However, despite these lower values, our data revealed an increment from 3.78 to 5.02 among children, with the equivalent values for the United Kingdom being 2.1 admissions per 100,000 person-years in 1998 to 9.2 admissions per 100,000 person-years in 2018 [5].

In the United States, the incidence of FIA increased by >3 times from 2004 to 2016 [12]. However, the percentage of FIA cases that required hospitalization decreased by 67% (2% in 2004 to 0.66% in 2016); therefore, the estimated incidence of admissions decreased from 1.73 to 1.58 per 100,000 person-years [12]. US data reported from the Kids’ Inpatient Database showed that for the population aged <20 years, the total annual hospitalization rates for FIA increased significantly from 1.15 per 100,000 person-years in 2006 to 1.53 per 100,000 person-years in 2012 (*P*<.001) [17].

On the basis of *ICD-10* codes, the frequency of hospitalization for FIA in Brazil underwent no significant changes between 2011 and 2019 [22], a trend that has been reported elsewhere [12,22,37].

To our knowledge, the only study conducted in Spain showed that hospital admissions for FIA increased significantly from 1998 to 2011 [26].

The epidemiology of FIA is not clearly understood, as shown by the fact that published estimates on the incidence of disease vary widely between countries [5,8,15,17,18,21,22,26,27,37], possibly because of limited access to and quality of medical data (including classification and coding issues), variations in definitions of anaphylaxis across countries, and changes over time in the local recommendations for management of FIA [5,8,15,17,18,21,22,26,27,37].

Several authors have attributed the marked reduction in the number of cases during 2020 to the COVID-19 pandemic [23,24,38]. This finding can be explained by decreased accidental exposures due to reduced social gatherings and eating out, school closures, and reluctance to visit the emergency department [23,24,38].

In our population, the male-to-female ratio among those aged <15 years was 1.63:1, decreasing to 1.07:1 among adults. These results coincide with those of other population studies, where prepubertal male overrepresentation was significant. This trend reversed from age 15 years onward [5-7,17,39].

We found that the mean age on admission decreased from 2016 to 2021, likely because of the pronounced increase in the incidence over time in the younger age group, as observed by other authors [8,40].

In our study, the types of foods responsible for FIA differed depending on the age group: “milk and dairy products,” “peanuts and tree nuts and seeds,” and “eggs” were more common among children, whereas “shellfish (crustaceans) and other fish,” “peanuts and tree nuts and seeds,” and “fruits and vegetables” were the most frequent triggers among adults. The analysis of 3427 cases of confirmed FIA included in the European Anaphylaxis Registry showed that the most frequent triggers of FIA in children were peanut, cow’s milk, cashew, and hen’s egg and that the most frequent triggers among adults were wheat flour, shellfish, hazelnut, and soy [6]. In line with our results, milk has been identified as the main food implicated in FIA in several European countries [7,8,26]. Food triggers vary by region and with country-specific consumption patterns [7,8,12,17,21,22,26,27,41].

Recently, Panagiotou et al [25] conducted a systematic review to assess the effects of the components of the Mediterranean diet on FAs. The results of this study indicate a generally positive association between adherence to the Mediterranean diet and the prevention of FAs. This finding is consistent with expectations, given the well-established health-promoting and anti-inflammatory characteristics of the Mediterranean diet. The diet is rich in beneficial nutrients such as polyphenols, n-3 long-chain polyunsaturated fatty acids, and other fat-soluble micronutrients [25].

The most frequently coded conditions among children hospitalized for FIA in Spain from 2016 to 2021 were atopic dermatitis (224/1535, 14.59%), followed by asthma (197/1535, 12.83%); in adults, the 3 most frequent conditions were hypertension (156/626, 24.9%), asthma (115/626, 18.4%), and diabetes mellitus (92/626, 14.7%).

In a recent systematic review and meta-analysis, Christensen et al [42] found that atopic dermatitis was common in individuals with FAs (pooled prevalence, 45.3%) and that individuals with FAs had a 4- to 5-fold higher risk of presenting atopic dermatitis than those without FAs. As in our investigation, this association was stronger for children [42]. However, comparison with other investigators is difficult, as the presence of concomitant atopic dermatitis seems to differ according to the food trigger, being higher in patients with anaphylaxis induced by hen’s egg and lower in patients with anaphylaxis induced by shellfish [6].

The prevalence of asthma in children and adults hospitalized for or treated for FIA in the emergency department is higher than that in the general population, both in Spain and in other countries [6,7,17,18,21,24,43,44].

Regarding the symptoms associated with FIA found in our study, children more frequently presented gastrointestinal symptoms and urticaria and less frequently presented acute respiratory failure, hypotension, and syncope than adults. This difference in distribution has been reported elsewhere [6,7]. Tanno et al [7] reported that skin, respiratory, gastrointestinal, and cardiovascular symptoms were the main clinical presentations among patients with FIA, although a detailed clinical description of the manifestations is often missing, consistent with other national database studies [6,16-18,21,22,24,26,37,41,43].

A total of 11 patients died after being hospitalized for FIA during the study period, that is, 0.51% (11/2161) of all admissions in Spain. The case fatality rate was very similar in the United Kingdom from 1998 to 2018, namely, 0.49% (152/30,700), decreasing from 0.70% in 1998 to 0.19% in 2018 [5]. Low fatality rates among patients hospitalized for FIA have been reported in other countries [14,18,41,45].

A recent systematic review found lower mortality rates for FIA than for anaphylaxis caused by other triggers [14]. However, between 2011 and 2019 in Brazil, IHM after admission for FIA was 4.36%, possibly because of a lack of AAIs and admission of more severe cases than in other countries. In any case, food was the least frequent cause of death among anaphylaxis admissions [22].

Using our definition of severe cases (admitted to ICU or fatal outcome), we observed figures ranging from 7% to 17% (global 11.99%), with the lowest value reported for the year 2021 (33/427, 7.7%). Data from the European Anaphylaxis Registry show that approximately 6% of cases were treated in the ICU [6] and that the frequency of admission to the pediatric ICU with anaphylaxis triggered by cow’s milk and hen’s egg among children aged ≤12 years did not exceed 5% [46]. Including all possible triggers, our proportion for the 0- to 14-year age group was 6.38%. In Japan, from 2016 to 2020, only 10 (0.74%) of 1344 children with FIA were treated in the ICU [47]. Variations in the use of AAIs, causative foods, or hospital protocols for referral to the ICU could justify these differences between countries [6,18,47]. However, the high rates of ICU admission found in our study require further investigation.

After multivariable adjustment, we found that belonging to the younger age group (0 to 14 years) was associated with less severe FIA, whereas a diagnosis of asthma or ischemic heart disease recorded in the discharge report was associated with more severe FIA.

Several authors have reported a higher incidence of less severe FIA in young children. However, the greatest risk of severe and fatal cases appears to be in adolescents and young adults, persisting well into the fourth decade of life [5,10,13,15,18]. High-risk behaviors, such as deliberately eating risky food or refusing to carry rescue medication, account for the greater severity reported among adolescents [16].

Turner et al [16] reviewed studies assessing the relationship between the severity of FIA and asthma, finding contradictory results. The meta-analysis showed no significant associations between the severity of FIA and previous asthma [16]. The authors concluded that although evidence is lacking, the degree of asthma control may be more relevant than a diagnosis of asthma [16]. There is a need for studies with detailed information on current treatment and control of asthma to clarify this association [6,16,18].

Using hospital data recorded between 1997 and 2011 in Spain, Nieto-Nieto et al [28] reported that being aged ≥50 years was associated with more severe all-cause anaphylaxis, possibly because of the effects of comorbid conditions. Furthermore, a study of 38,000 hospital admissions for all-cause anaphylaxis conducted in the United States revealed that among the 11.6% of cases considered severely ill (eg, intubation, ICU admission, or near‐fatal reaction), the predictors of severity included medication as a trigger, age >65 years, and the presence of cardiac and comorbid respiratory conditions [48]. We agree with the results of these investigations because ischemic heart disease was an independent predictor of severity among the adults in our study population.

The huge repercussions that FIA has on the lives of patients who are affected and on the health system necessitate strategies to reduce their magnitude and impact [3,7,8,11,18].

It is important to improve the level of knowledge and alertness in both the public and patients who are affected through campaigns in the media or on social networks in collaboration with health and educational organizations. It is essential that all persons with known FAs receive personalized recommendations about their disease, prevention, and management of AAIs to avoid and control possible accidental exposures. New technologies must be evaluated and introduced to improve the management of people with FAs [49].

Desensitization should be considered in patients at high risk of severe FIA. Persons with asthma must improve control of their disease to avoid having a severe condition if they experience FIA [3,7,8,11,18].

The relevant authorities should improve prevention strategies in food and catering areas, including correct labeling with adequate information on both packaged and unpackaged food, together with a list of notifiable ingredients [18].

From an epidemiological perspective, it is advisable to improve and expand the surveillance, registration, classification, and coding of FIA [18]. Several recommendations have been put forward in this regard: (1) the promotion of networking and large-scale registries on anaphylaxis (both food and nonfood triggers) to enable detailed analysis of reactions; (2) further research into risk factors and potential biomarkers for predicting severity; and (3) adoption of the new *International Classification of Diseases, Eleventh Revision* (*ICD-11*) classification of allergic and hypersensitivity reactions [7,18]. It is expected that *ICD-11* will be implemented in Spain in the coming years. This new coding method will enhance the collection of more reliable, accurate, and comprehensive epidemiological data on FIA. The data collected will support the quality management of patients with FAs and FIA and facilitate better health care planning, decision-making, and implementation of public health measures to prevent and reduce the morbidity and mortality associated with these conditions. The improved logic and standardized definitions provided by *ICD-11* will also streamline international comparisons of quality care and facilitate global sharing of best practices [7,8,50].

The main strength of our investigation is that we analyzed a national sample of real-world data that included >95% of patients admitted to public and private hospitals across Spain. Given that we provide representative data for a whole country over a 6-year period, we can draw robust conclusions on the characteristics of hospitalizations due to FIA and on changes in these characteristics over time [24,26,28,29,43].

The other strengths of our study include standardized data acquisition using *ICD-10* codes and our comprehensive assessment of the main chronic conditions associated with FIA outcomes.

### Limitations

However, our findings are subject to limitations. First, the validity of *ICD-10* codes for FIA has not been assessed in the CMBD. Validation studies from the United States, Germany, and Taiwan for all-cause anaphylaxis reported positive predictive values (PPVs) of 60% to 65% [51-53]. The authors suggest that increasing the accuracy of definitions by restricting research to primary discharge codes may improve the PPVs but reduce sensitivity [51-53]. In our opinion, even if a moderate PPV had been recorded in our study, using the same database and methods throughout the study period, we would still be able to monitor time trends irrespective of the risk of miscoding, which is unlikely to have been affected by time. However, because our results may have been affected by changes in coding practices over time, further investigations are required to improve the accuracy of anaphylaxis coding to ensure correct patient identification in health care databases. Second, the CMBD does not include patients with FIA treated in the emergency department who did not require hospital admission, with the result that the burden of FIA in the health system is underestimated. Third, given that the severity of FIA and other conditions is not recorded in our database, severity was assessed using admission to intensive care or fatal outcomes as a proxy, as suggested by other authors [6,18,47]. Fourth, hospital-based databases such as the one used in this study are affected by a relevant proportion of uncoded, “unspecified” causes of FIA [5,6,10,21,22,26,27]. Fifth, as described in other population‐based studies, it was not possible to identify recurrences or biphasic cases of FIA; therefore, most epidemiological studies using incidence as the main variable may overestimate the number of anaphylactic episodes [51-53]. Sixth, cofactors not collected in the database included alcohol consumption, exercise, viral infections, drugs (eg, nonsteroidal anti-inflammatory drugs, β‐blockers, and angiotensin-converting enzyme inhibitors), and sleep deprivation, which have been reported to play a role in FIA reactions and severity [6,16,18,54]. Seventh, data on sensitization profiles, use of AAIs, and other treatments are missing from the database.

### Conclusions

In Spain, IHM and the incidence of hospitalizations due to FIA are low and remain stable over time, with a small but significant increment in the number of cases only among children. Findings for food as a trigger and baseline characteristics differ between children and adults. The proportion of severe cases is high and did not improve from 2016 to 2021, with older age and asthma being risk factors for severity. It is mandatory to improve the surveillance of FIA and to implement preventive strategies to reduce the burden of FIA.

## Appendix

Multimedia Appendix 1 International Classification of Diseases, Tenth Revision (ICD-10) codes used to identify food-induced anaphylaxis, clinical conditions, and procedures.

Multimedia Appendix 2 International Classification of Diseases, Tenth Revision (ICD-10) codes used as primary diagnosis codes when food-induced anaphylaxis was coded as a secondary diagnosis.

Multimedia Appendix 3 Multivariable analysis to identify variables associated with severe anaphylaxis during a hospital admission with a diagnosis of food-induced anaphylaxis in Spain between 2016 and 2021, according to age.

## Abbreviations

AAI: adrenaline autoinjector
CMBD: Conjunto Minimo Basico de Datos (Spanish National Registry of hospital discharges)
FA: food allergy
FIA: food-induced anaphylaxis
ICD-10: International Classification of Diseases, Tenth Revision
ICD-11: International Classification of Diseases, Eleventh Revision
ICU: intensive care unit
IHM: in-hospital mortality
OR: odds ratio
PPV: positive predictive value

## Data Availability

The data sets generated during and analyzed during this study are not publicly available due to the Spanish Ministry of Health requires the investigators to accept the following obligations before transfer of the data: (1) to treat all information under strict confidentiality conditions, (2) not to use and not to authorize any natural or legal person to use the transferred data other than exclusively for the purposes of the work, as reflected in the request, (3) to destroy the file or data provided and all copies made of it once the period for which the data are required has elapsed. Access to the data from the Spanish Ministry of Health can be requested [55].
