# Supplementary material for: Hospitalizations for Food-Induced Anaphylaxis Between 2016 and 2021: Population-Based Epidemiologic Study
Source: JMIR Public Health Surveill. 2024 Aug 27;10:e57340. doi: 10.2196/57340 (PMC11387914; doi:10.2196/57340)
Supplement: Multimedia Appendix 1 [file publichealth_v10i1e57340_app1.doc]

Multimedia Appendix 1. ICD-10 codes used to identify food induced anaphylaxis, clinical conditions, and procedures.

| **Variable** | **ICD-10** |
| --- | --- |
| Unspecified food | T78.00 |
| Peanuts | T78.01 |
| Shellfish (crustaceans) | T78.02 |
| Other fish | T78.03 |
| Fruits and vegetables | T78.04 |
| Tree nuts and seeds | T78.05 |
| Food additives | T78.06 |
| Milk and dairy products | T78.07 |
| Eggs | T78.08 |
| Other food products | T78.09 |
| Asthma | J45 |
| Obesity | E66 |
| GERD | K21 |
| Chronic rhinitis | J31.0 |
| Atopic dermatitis | L20 |
| Anxiety | F41 |
| Depression | F32 |
| COPD | J44 |
| Hypertension | I10 |
| Ischemic heart disease | I21 |
| Atrial fibrillation | I48 |
| Hypothyroidism | E03 |
| Hyperthyroidism | E05 |
| Diabetes mellitus | E11 |
| Hypotension | I95 |
| Syncope/collapse | R55 |
| Nausea/vomiting | R11.2 |
| Abdominal pain | R10 |
| Acute respiratory failure | J96.0 |
| Urticaria | L50 |
| Noninvasive mechanical ventilation | 5A09357, 5A09457, 5A09557 |
| Invasive mechanical ventilation | 5A1945Z, 5A1955Z, 5A1935Z |

GERD: Gastroesophageal reflux disease; COPD: chronic obstructive pulmonary disease
