# Supplementary material for: Hospitalizations for Food-Induced Anaphylaxis Between 2016 and 2021: Population-Based Epidemiologic Study
Source: JMIR Public Health Surveill. 2024 Aug 27;10:e57340. doi: 10.2196/57340 (PMC11387914; doi:10.2196/57340)
Supplement: Multimedia Appendix 2 [file publichealth_v10i1e57340_app2.doc]

Multimedia Appendix 2. ICD-10 codes used as primary diagnosis code when food induced anaphylaxis was coded as a secondary diagnosis.

| CODIGO | N | % |  |
| --- | --- | --- | --- |
| T78.1 | 321 | 45.1 | Other adverse food reactions, not elsewhere classified |
| T78.3 | 18 | 2.5 | Angioneurotic oedema |
| Z01.82 | 9 | 1.3 | Encounter for allergy testing |
| Z51.6 | 9 | 1.3 | Encounter for desensitization to allergens |
| L50.0 | 8 | 1.1 | Allergic urticaria |
| J98.01 | 7 | 1.0 | Acute bronchospasm |
| T78.2 | 6 | 0.8 | Anaphylactic shock, unspecified |
| J96.00 | 5 | 0.7 | Acute respiratory failure, unspecified whether with hypoxia or hypercapnia |
| L27.2 | 5 | 0.7 | Dermatitis due to ingested food |
| J96.01 | 3 | 0.4 | Acute respiratory failure with hypoxia |
| R09.02 | 2 | 0.3 | Hypoxemia |
| J04.0 | 1 | 0.1 | Acute laryngitis |
| J96.20 | 1 | 0.1 | Acute and chronic respiratory failure, unspecified whether with hypoxia or hypercapnia |
| J96.90 | 1 | 0.1 | Respiratory failure, unspecified, unspecified whether with hypoxia or hypercapnia |
| J96.91 | 1 | 0.1 | Respiratory failure, unspecified with hypoxia |
| J96.92 | 1 | 0.1 | Respiratory failure, unspecified with hypercapnia |
| L50.5 | 1 | 0.1 | Cholinergic urticaria |
| L53.8 | 1 | 0.1 | Other specified erythematous conditions |
| R57 | 1 | 0.1 | Shock, not elsewhere classified |
| R60.0 | 1 | 0.1 | Localized edema |
| T61.8 | 1 | 0.1 | Toxic effect of noxious substances eaten as seafood |
