# Supplementary material for: Hospitalizations for Food-Induced Anaphylaxis Between 2016 and 2021: Population-Based Epidemiologic Study
Source: JMIR Public Health Surveill. 2024 Aug 27;10:e57340. doi: 10.2196/57340 (PMC11387914; doi:10.2196/57340)
Supplement: Multimedia Appendix 3 [file publichealth_v10i1e57340_app3.doc]

Multimedia Appendix 3. Multivariable analysis to identify variable associated with severe anaphylaxis during a hospital admission with a diagnosis of food induced anaphylaxis in Spain (2016-2021) according to age.

| **Children (0-14 years)** | | | **Adults (15+ years)** | | | **All ages** | | |
| --- | --- | --- | --- | --- | --- | --- | --- | --- |
| **Variables** | | **OR (95%CI)** | **Variable** | | **OR (95%CI)** | **Variable** | | **OR (95%CI)** |
| Age, years | 0-4 | 1 | Age, years |  | NA | Age, years | 0-14 | 1 |
|  | 5-9 | 1.56(0.92-2.68) |  | 15-59 | 1 |  | 15-59 | 5.1(3.11-8.36) |
|  | 10-14 | 1.34(0.75-2.4) |  | 60+ | 1.34(0.79-2.26) |  | 60+ | 3.87(1.99-7.53) |
| Asthma | | - | Asthma | | - | Asthma | | 1.71(1.12-2.58) |
| Ischemic heart disease | | - | Ischemic heart disease | | 2.25(1.48-3.68) | Ischemic heart disease | | 1.89(1.15-3.16) |
| Hypotension | | 4.17(1.38-12.6) | Hypotension | | - | Hypotension | | - |
| Acute respiratory failure | | 5.06(2.27-11.3) | Acute respiratory failure | | 4.35(2.38-7.93) | Acute respiratory failure | | 4.62(2.89-7.41) |

OR Odds ratio. CI Confidence interval
